# The Glyphosate-Based Herbicide Roundup Does Not Elevate Genome-Wide Mutagenesis of *Escherichia coli*

**DOI:** 10.1534/g3.117.300133

**Published:** 2017-08-09

**Authors:** Clayton Tincher, Hongan Long, Megan Behringer, Noah Walker, Michael Lynch

**Affiliations:** Department of Biology, Indiana University, Bloomington, Indiana 47405

**Keywords:** ecological dependence of mutations, evolutionary genomics, environmental mutagenesis, mutagenicity test, herbicide damage

## Abstract

Mutations induced by pollutants may promote pathogen evolution, for example by accelerating mutations conferring antibiotic resistance. Generally, evaluating the genome-wide mutagenic effects of long-term sublethal pollutant exposure at single-nucleotide resolution is extremely difficult. To overcome this technical barrier, we use the mutation accumulation/whole-genome sequencing (MA/WGS) method as a mutagenicity test, to quantitatively evaluate genome-wide mutagenesis of *Escherichia coli* after long-term exposure to a wide gradient of the glyphosate-based herbicide (GBH) Roundup Concentrate Plus. The genome-wide mutation rate decreases as GBH concentration increases, suggesting that even long-term GBH exposure does not compromise the genome stability of bacteria.

Glyphosate—N-(phosphonomethyl)glycine—kills sensitive plants by inhibiting the enzyme 5-enolpyruvylshikimate-3-phosphate synthase and thus blocking the synthesis of aromatic amino acids such as tryptophan, tyrosine, and phenylalanine (Steinrücken and Amrhein 1980). GBHs, such as Roundup, are the most heavily used herbicides worldwide. Over the last decade, the use of GBHs has increased by ∼60%, because of the introduction of genetically-engineered herbicide-tolerant crops, the drop in price of glyphosate, and the emergence of resistant weeds (Benbrook 2016; Myers *et al.* 2016). In 2014, the total volume of glyphosate applied worldwide was enough to treat between 22 and 30% of globally cultivated cropland, a figure higher than any other pesticide in history (Benbrook 2016).

As GBHs have become the herbicide of choice, the concern over their impact on the environment through soil leaching and water runoff has also risen. Although some studies suggest that glyphosate absorbs quickly into the soil, reducing its availability to impact biological systems, recent studies have raised the concern that the half-life in soil is longer than previously thought (Borggaard and Gimsing 2008; Myers *et al.* 2016). Additional concerns have been raised regarding the levels of glyphosate residue found in commercial foods and their potential impact on mammalian kidney and liver cells (Myers *et al.* 2016). In 2015, the World Health Organization’s International Agency for Research on Cancer determined that glyphosate was “probably carcinogenic to humans,” noting DNA damage and limited evidence of an increase of non-Hodgkin lymphoma in heavily applied areas (IARC Working Group 2015). However, these findings differ from those of Rank *et al.* (1993) and Dimitrov *et al.* (2006), which found no evidence of chromosomal aberrations in mammalian cells, as well as the evaluation on glyphosate by the European Food Safety Authority (Portier *et al.* 2016).

Although many studies have been done on the acute toxicity and mutagenicity of glyphosate and GBHs, most of them tend to focus on the impact of short-term exposure to compounds, rather than the effects of consistent long-term exposure, or indirectly evaluate mutagenic effects based on phenotypic response (Bolognesi *et al.* 1997; Raipulis *et al.* 2009; Rank *et al.* 1993; Li and Long 1988). These studies often suffer from mixed results, and usually depend on the experimental model or do not use a commercial formulation (such as Roundup Concentrate Plus). The greater effect shown by commercial formulations is attributed to additives and surfactants, such as diquat dibromide and polyoxyethylenamine, or a synergistic effect between the additives and glyphosate (Myers *et al.* 2016; Rank *et al.* 1993; Bolognesi *et al.* 1997). Short-term studies also ignore the potential for multiple applications throughout the growing season and the possibility of residual levels of the herbicide in soil and water. To our knowledge, no other experiments have been conducted that focus on the long-term mutagenic effects of chronic exposure on the genome of a nontarget organism. The ubiquitous use of GBHs, as well as the new data on soil half-life and the potential for runoff, along with the mixed results of various toxicity and mutagenesis studies, highlights the need for an unbiased method to measure the mutagenic impact of GBHs on nontarget organisms in the environment.

WGS of lines produced in MA experiments can be used to evaluate mutagenesis of organisms under long-term pollutant exposure (Long *et al.* 2016). MA experiments allow all but the most deleterious mutations to accumulate in the lines by applying single-cell bottlenecks to initially identical lines, and WGS of the final MA lines directly reveals the mutations (Bateman 1959; Mukai 1964; Kibota and Lynch 1996; Lynch *et al.* 2008). This MA/WGS procedure has been successfully applied to a variety of prokaryotic and eukaryotic organisms, yielding accurate estimates of the genome-wide rate and molecular spectrum of spontaneous mutations [reviewed in Lynch *et al.* (1999) and Halligan and Keightley (2009)]. Among these model organisms, *Escherichia coli* is a particularly useful and accessible genetic model to study the patterns and mechanisms of mutational response to long-term exposure to pollutants using the MA/WGS method (Long *et al.* 2016; Kibota and Lynch 1996). Most MA/WGS studies are currently limited to spontaneous mutations, with Long *et al.* (2016) being the first study to apply a potential mutagen into the system.

In this study, we apply the MA/WGS procedure to evaluate GBH’s effects on the rate and molecular spectrum of genomic mutations in two sets of *E. coli* MA lines exposed to a gradient of sublethal Roundup Concentrate Plus concentrations for 2–3.5 months. Two strains of *E. coli* were utilized in this experiment: wild-type K-12 MG1655 and a postreplicative DNA mismatch-repair (MMR)-deficient mutator strain. The first is a widely-used type strain with a characteristic background mutation rate for a prokaryote (Lee *et al.* 2012; Lynch *et al.* 2016), while the latter has an increased mutation rate due to an engineered deletion of the *mutS* gene, which inhibits the strain’s ability to remove premutations. Mutations accumulated in the repair-deficient strain are untouched by postreplication DNA MMR (one of the most powerful repair pathways for removing premutations). Therefore, comparison of MA-line mutation rates between both strains under GBH treatments may reveal whether GBH compromises MMR function.

## Materials and Methods

### Strains, media, and transfers

The wild-type (laboratory designation name, PFM2) and Δ*mutS* (PFM342) strains were in the K-12 MG1655 background and provided by Patricia Foster’s lab, Indiana University. Roundup Concentrate Plus was ordered from The Home Depot (model No.: 5005001; recommended usage concentration by the manufacturer: 3200 ppm). Difco LB agar (BD, catalog number: 244510; a rich medium containing yeast extract, peptone, NaCl, and agar) was used for culturing MA lines. The agar surface pH was measured using a Ross flat-bottom pH electrode.

Single-colony daily transfers were done on each MA line, treated with gradients of Roundup Concentrate Plus for 2–3.5 months (Table 1).

**Table 1:** Details of MA lines grown at different GBH concentrations

Strains	Conc.	Group	n	Tr	pH	Divisions	Ts	Tv	Ins	Del
+	0	A	28	99	7.25	2427	48	33	3	8
+	6.4	D	25	97	7.23	2350	46	37	1	4
+	51.2	E	27	98	7.21	2441	27	41	4	7
+	409.6	F	25	88	7.18	2050	28	20	1	8
+	3276.8	G	23	87	7.01	1931	15	21	0	3
+	26214.4	H	45	62	5.92	1414	17	20	1	6
Δ*mutS*	0	SA	8	59	7.25	1410	1243	30	131	110
Δ*mutS*	6.4	SD	10	55	7.23	1304	1380	97	130	125
Δ*mutS*	51.2	SE	9	56	7.21	1322	1168	34	91	80
Δ*mutS*	409.6	SF	5	88	7.18	2050	997	31	98	86
Δ*mutS*	3276.8	SG	7	87	7.01	1992	1160	92	98	99
Δ*mutS*	26214.4	SH	9	62	5.92	1420	593	21	83	64

Conc., glyphosate-based herbicide concentration in parts per million; Group, group label for samples under the treatment; n, total number of MA lines in the final analyses; Tr, mean number of transfers each MA line experienced; pH, mean pH value measured from agar surface; Divisions, mean number of cell divisions each MA line passed; Ts, total number of transitions in the group; Tv, total number of transversions; Ins, total number of insertions in the group; Del, total number of deletions detected in the group.

### Efficiency of plating (EOP) upon GBH treatment

Cells were cultured for ∼17 hr on a tissue rotator. Culture was then serially diluted and ∼1500 cells were plated onto LB plates with 0, 6400, 12,800, 25,600, 51,200, and 102,400 ppm Roundup Concentrate Plus with five replicates for each concentration. Colony-forming units (CFU) were counted after 24 hr at 37°. EOP was calculated by dividing CFU of the GBH plates with that of the blank control. This assay was repeated three times at different time points.

### Experimental design and transfers

Six groups of wild-type cell lines treated with an 8× progression of Roundup Concentrate Plus concentrations (0, 6.4, 51.2, 409.6, 3276.8, and 26,214.4 ppm) were all initiated from a single wild-type colony. All groups had 32 replicates, except that the 26,214.4 ppm group had 48 replicates. A large number of replicates were used for each group to ensure that as many nonlethal mutations that arose as possible could accumulate in the cell lines. The 26,214.4 ppm group had more replicates than other groups because of the shorter amount of time that it was transferred (∼2 months *vs.* 3.5 months in other groups) (Table 1). All lines were single-colony transferred daily (24 hr) and incubated at 37°. Six more groups of Δ*mutS* lines treated with the above concentrations of Roundup Concentrate Plus were initiated from a single Δ*mutS* colony. The Δ*mutS* cell lines were transferred on the same schedule as the wild-type cell lines. All groups had 10 cell lines, while the Δ*mutS* group treated with the highest concentration had 16 due to the aforementioned shorter transfer time.

The number of cell divisions was estimated approximately every 3 wk. For each group, single colonies from ∼5 randomly-selected cell lines were cut from agar plates, serially diluted with 1× PBS, and plated onto plain LB agar plates. Assuming synchronized exponential growth, the cell division number was calculated by log_2_*N*, where *N* is CFU. The total cell division estimate passed for each line was calculated by taking the overall mean number of cell divisions between transfers throughout the entire experiment multiplied by the number of transfers.

### DNA extraction, library construction, and genome sequencing

The genomic DNA was extracted using the Wizard Genomic Purification Kit (Promega). We then constructed Illumina genome libraries using Nextera DNA Library Prep kit (Illumina catalog number: FC-121-1030) and applied Hiseq2500 150 bp paired-end WGS (Hubbard Center for Genome Studies, University of New Hampshire) to each final evolved MA line.

### Mutation analyses

We used Trimmomatic 0.32 (Bolger *et al.* 2014) to trim off adaptors and then mapped reads to the reference genome using BWA-0.7.10 mem (GenBank genome accession numbers: NC_000913.3) (Li and Durbin 2009). Duplicate reads were eliminated using picard-tools-1.141 and reads around indels were realigned using GATK-3.5 before preforming SNP and indel discovery with standard hard filtering parameters described by GATK Best Practices recommendations (Phred-scaled quality score QUAL > 100 and RMS mapping quality MQ > 59 for both variant and invariant sites) (McKenna *et al.* 2010; DePristo *et al.* 2011; Van der Auwera *et al.* 2013). We used UnifiedGenotyper in GATK to call base pair substitutions (BPS) and indels. Greater than 99% of reads were required in a line to determine the line-specific consensus nucleotide at a candidate site; 1% was set to allow for aberrant reads originated from sequencing errors, impure indexes during library construction, or barcode degeneracy during sequence demultiplexing. Mutation rate µ was calculated by $\mu =\left(m/\sum_{1}^{n}N\times T\right),$ where *m* is the total number of mutations pooled from all MA lines, *n* is the total number of lines, *N* is the analyzed sites in one line, and *T* is the number of cell divisions that the MA line passed.

MA lines with < 15× sequencing depth of coverage or exhibiting cross-contamination were removed from the final analyses. We identified cross-contamination by searching for shared mutations after genome sequencing (unless they are from mutation hotspots or genes under strong selection) especially in lines sharing the same culturing plate.

Insertions of IS (Insertion Sequence) elements through transpositions were analyzed using GRASPER (https://github.com/COL-IU/GRASPER), which constructed an Al-Bruijn graph (*l* = 90, error rate of 5%) based on the algorithms described in Lee *et al.* (2014). Coverage analyses for detecting large duplications and prophage deletions, as well as identification of false positives caused by such structural variants, followed Long *et al.* (2016).

### Statistics

All statistical analyses and plotting were done in R 3.1.3 (R Development Core Team 2015).

### Data availability

MA lines are available upon request. Supplemental Material, File S1, contains Table S1, Table S2, Table S3, Table S4 and Table S5. Table S1 contains details of mutations of MA lines under different treatments. Table S2 contains the list of detected BPS mutations. Table S3 contains the list of indels detected in this study. Table S4 contains results of mutation enrichment analysis on mutated genes. Table S5 contains information about IS element insertion rate in each treatment. All raw Illumina reads have been uploaded to the NCBI SRA under BioProject ID of PRJNA376412 (SRA study No. SRP100616).

## Results and Discussion

**Figure 1:**
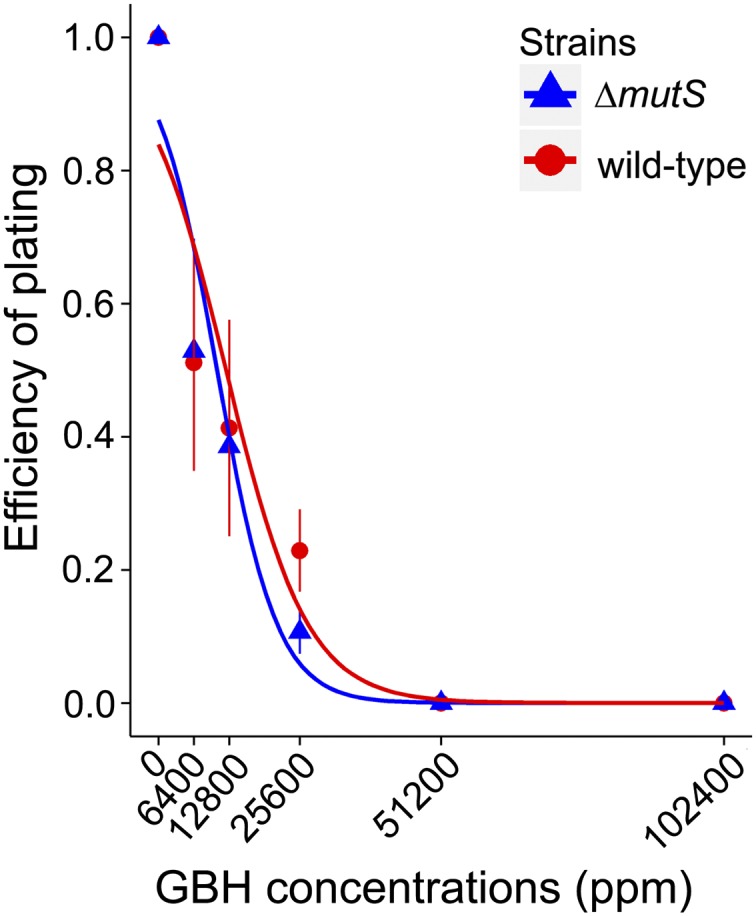
Efficiency of plating of the two strains treated with Roundup Concentrate Plus. The plotted lines are logistic regressions. Error bars are SEM. GBH, glyphosate-based herbicide.

We investigated the genome-wide mutagenic effects of Roundup Concentrate Plus after first determining the range of applicable sublethal concentrations of the herbicide. The two progenitor strains were exposed to a large gradient of GBH, plating ∼1500 cells on LB plates, and incubating at 37° for 24 hr, and the EOP was calculated by dividing the number of CFU of GBH plates with that of plain LB plates. The EOP, which estimates the fraction of cells not inhibited by the herbicide, decreased with increasing GBH concentration in both strains (Figure 1).

From a single progenitor cell of the wild-type strain, we created six groups of MA lines (0, 6.4, 51.2, 409.6, 3276.8, and 26,214.4 ppm of Roundup Concentrate Plus) and grew 32–48 replicate lines treated on LB plates with a daily single-colony transfer procedure. Similarly, 10–16 MA lines were established from a single Δ*mutS* progenitor cell for each of the above GBH concentrations. After about 2–3.5 months of daily single-colony transfers of these MA lines, we sequenced whole genomes and analyzed mutations in each of the final evolved lines. Details of the MA lines are shown in Table 1, Table S1, Table S2, and Table S3.

We also annotated the functional context of BPS (Table S2) of each treatment. None of their nonsynonymous/synonymous BPS ratios significantly differed from that of the control (χ^2^ test, *P* > 0.05 in all cases). The majority of BPSs are thus not selectively biased by GBH treatment but simply accumulate in a near-neutral fashion.

**Figure 2:**
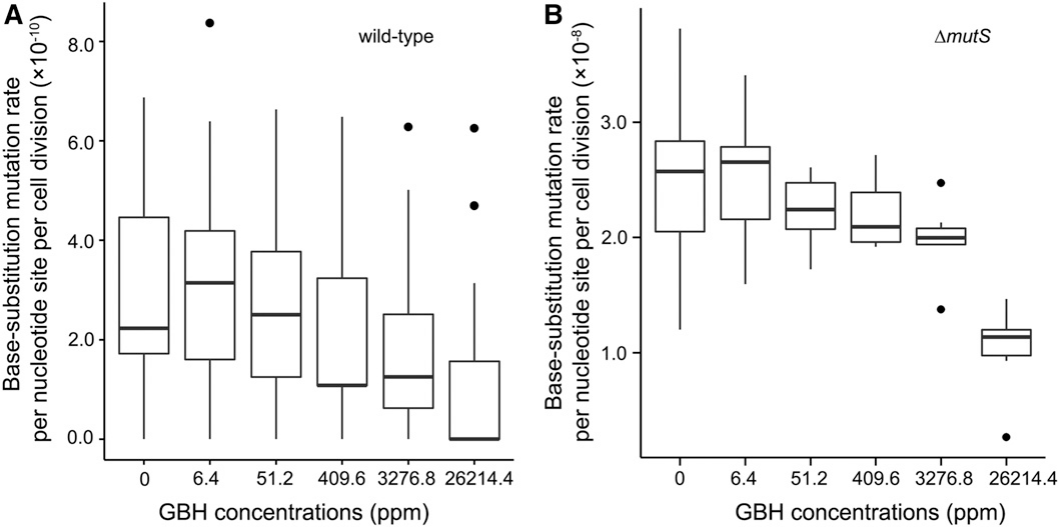
Base pair substitution mutation rates of the wild-type (A) and Δ*mutS* (B) strains at different Roundup Concentrate Plus concentrations. Black dots are outliers, which are determined by being greater than Q3 + 1.5 × IQR or lower than Q1−1.5 × IQR, where Q3 is the third quartile, Q1 is the first quartile, and IQR is the distance between the first and the third quartile (McGill *et al.* 1978). GBH, glyphosate-based herbicide; IQR, interquartile range; ppm, parts per million; Q, quartile.

**Figure 3:**
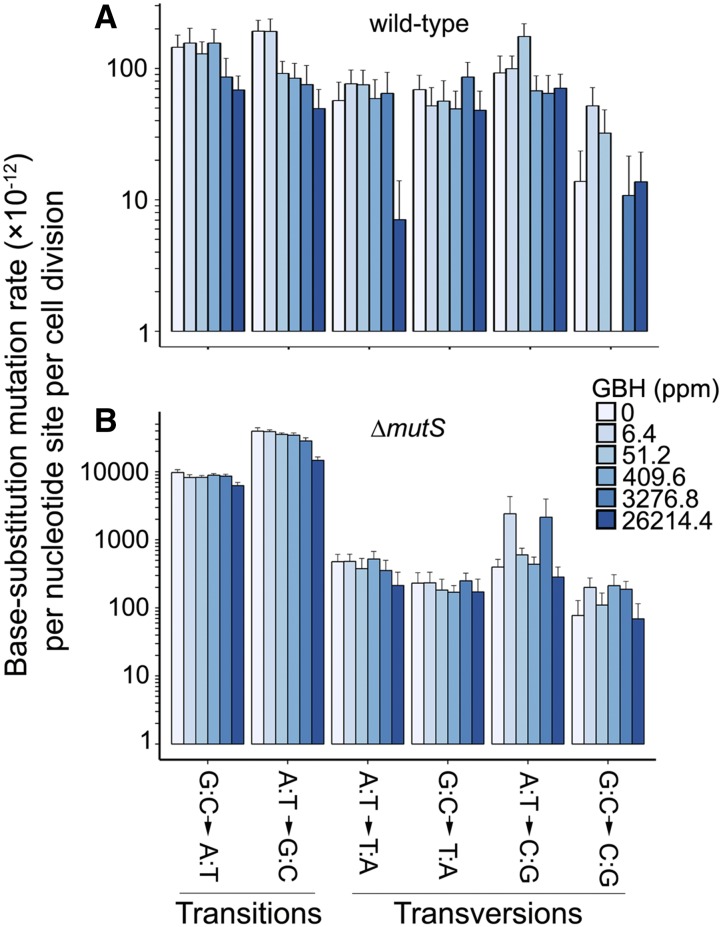
Mutation spectra of the wild-type and Δ*mutS* strains at different GBH concentrations. (A) Correlation parameters of wild-type mutation rate *vs.* GBH concentration: for G:C→A:T transition mutation rate, Pearson’s coefficient *r* = −0.78, *P* = 0.07 and A:T→G:C transition mutation rate, *r* = −0.55, *P* = 0.25. (B) Correlation parameters of Δ*mutS* mutation rate *vs.* GBH concentrations: G:C→A:T, *r* = −0.88, *P* = 0.02 and A:T→G:C, *r* = −0.95, *P* = 0.004. Error bars are SEM. GBH, glyphosate-based herbicide; ppm, parts per million.

BPS mutation rates show a strongly negative correlation with GBH concentrations that is marginally significant (Pearson’s correlation coefficient *r* = −0.81, *P* = 0.05; Figure 2A). Such correlation is stronger and significant when the DNA MMR is dysfunctional, *i.e.*, in Δ*mutS* MA lines (*r* = −0.96, *P* = 0.002; Figure 2B). Because Roundup Concentrate Plus is acidic and increasing hydrogen ions might improve replication fidelity of DNA polymerases (Eckert and Kunkel 1993), the above negative correlations could be an outcome of pH change by the increase of GBH doses. GBH treatment does not elevate any specific types of mutations either, but decreases the mutation rate of transitions, especially in Δ*mutS* lines (Figure 3). The ratio of mutation rates in MMR^+^ and MMR^−^ lines measures the proportion of premutations not eliminated by MMR and does not correlate with GBH concentration either (*r* = −0.01, *P* = 0.98). This suggests that the MMR function may not be affected by the GBH treatment, unlike other chemicals such as antimicrobial agents (Long *et al.* 2016; Gutierrez *et al.* 2013).

Although we do not detect significant correlations between the mutation rate and GBH treatment, there is still the chance that mutations are enriched in certain genes. Thus, we pooled the BPS mutations in the coding regions for all genes in the wild-type lines treated with GBH (205 BPSs in 196 genes). Then, for each gene, we calculated the Poisson probability of seeing greater than or equal to the number of observed mutations in the gene, given the expected mutation rate of the gene with GBH treatment (Table S4). After Bonferroni correction, we do not find any genes enriched with mutations. Thus, under the current rich medium-culturing environment, GBH does not elevate mutations of any particular genes either.

We also analyzed the transposition of IS elements, the simple transposable elements in *E. coli*, and did not find any significant correlation with GBH dose (for wild-type, *r* = 0.64, *P* = 0.17; for Δ*mutS*, *r* = −0.005, *P* = 0.93; Table S5, all Poisson confidence intervals overlap between different treatments). No significant elevation of other structural variants such as chromosomal duplication and prophage deletion by GBH treatment is observed; thus, GBH does not promote structural variant mutations either. However, this does not indicate that GBH is not harmful to bacteria; instead, bacteria are inhibited especially at higher concentrations (Figure 1), though such inhibition is not at the DNA-level.

### Conclusions

Our study demonstrates a “worst case” scenario, wherein the test organism is continuously subjected to a constant level of GBH with no charged soil particles available for absorption of glyphosate or other chemicals in the herbicide. Surprisingly, even months of growth under a very high GBH dose did not elevate genome-wide mutagenesis in *E. coli*, based on thousands of directly detected and unbiased mutations, ranging from single BPS to large structural variants. We thus conclude that GBH is not mutagenic to bacteria.

## Supplementary Material

Supplemental material is available online at www.g3journal.org/lookup/suppl/doi:10.1534/g3.117.300133/-/DC1.
